# Editorial: Computational intelligence for signal and image processing

**DOI:** 10.3389/fncom.2023.1284600

**Published:** 2023-09-27

**Authors:** Deepika Koundal, Baiyuan Ding

**Affiliations:** ^1^AI Cluster, School of Computer Science, University of Petroleum and Energy Studies, Dehradun, India; ^2^Science and Technology on Automatic Target Recognition Laboratory, National University of Defence Technology, Chansha, China

**Keywords:** image processing, signal processing, machine learning, deep learning, computational techniques

## 1. Introduction

The contemporary world features an array of sensors, each with distinct functions. Data from these sensors primarily come in the form of signals, images, videos, and similar formats (Cheng D. et al., 2022). Effectively deciphering this data holds the key to enhancing daily life and industrial efficiency (Wang et al., 2023). Initially, humans were responsible for processing and interpreting signal and image data, a process with limited accuracy and efficiency (Liu F. et al., 2023). However, the evolution of computational intelligence, including machine learning and deep learning, has enabled the automated handling of sensor measurements, reducing the need for human involvement (Jiang et al., 2023). Consequently, vast amounts of signal and image data can be efficiently processed for diverse applications (Cheng L. et al., 2022; Wang et al., 2022; Fu et al., 2023), given their varied and abundant nature, which encompasses radar signals, biomedical signals, optical images, and distinctive medical images (Zhuang et al., 2022a). To this end, distinct computational intelligence algorithms are necessary for various signal and image types (Zhuang et al., 2022b; Dang et al., 2023; Lu et al., 2023). Recent strides in machine learning and deep learning have introduced a suite of tools for signal and image processing like convolutional neural networks, deep belief networks, and deep generative models (Liu et al., 2021). Integrating these pioneering computational intelligence techniques into the realm of signal and image processing holds the promise of delivering accurate and rapid interpretations (Cong et al., 2023; Liu H. et al., 2023).

## 2. Contributions

Within this research domain, a total of 10 articles have been published. Pan et al. introduced a stepped image semantic segmentation network structure that incorporated a multi-scale feature fusion scheme and boundary optimization. It enhanced the model accuracy by optimizing the spatial pooling pyramid module in the Deeplab V3+ network by employing the Funnel ReLU activation function for accuracy improvement. Experimental results have shown that the enhanced networks achieved a 96.35% accuracy. Furthermore, Zhijian et al. explored a method for simulating the infrared data, fusing simulated 3D infrared targets with real infrared images. Real infrared images were fused into panoramic backgrounds, simulating infrared characteristics on aircraft components like the tail nozzle, skin, and tail flame. This approach, driven by Unity3D, allowed flexible aircraft trajectory and attitude editing, generating diverse multi-target infrared data. The experimental results have shown that the simulated image closely resembled the real infrared images and aligned with real data's target detection algorithm performance. Another study by Prabhakar et al. focused on EEG signal modeling and classification. With a sparse representation model and sparseness measurement analysis for EEG signals, Swarm Intelligence (SI) techniques were harnessed for Hidden Markov Model (HMM)-based classification. Additionally, a Convolutional Neural Network (CNN)-powered deep learning methodology achieved a remarkable 98.94% classification accuracy.

Additionally, Fan et al. have given insights to elucidate the association between Tic disorder and gut microbiota. A total of 78 stool samples were examined from Tic disorder cases and 62 from healthy controls, utilizing a case-control design for all studies. The results have shown variations in gut microbiota taxonomy between Tic disorder cases and controls, albeit with inconsistencies across studies. In another study, Saikumar et al. integrated the Internet of Things sensor data into a deep learning-based application for diagnosing heart conditions. The Internet of Things sensor data related to heart disease was utilized to train the deep graph convolutional network (DG_ConvoNet). The K-means technique was employed to reduce sensor data noise, aiding the clustering of unstructured data. Extracted features were then used in Linear Quadratic Discriminant Analysis. DG_ConvoNet, a deep learning approach, exhibited 96% accuracy, 80% sensitivity, 73% specificity, 90% precision, 79% F-Score, and a 75% area under the ROC curve, proficiently classifying and predicting heart ailments. Furthermore, Yan et al. have discussed urban street color analysis schemes by merging the color cards with efficient software recognition by addressing the challenges in quantifying urban color research. Using the China Building Color Card and Python's HSV color segmentation, Avenida de Almeida Ribeiro's colors from various angles have been assessed. This approach combined color card colorimetry and computer recognition by capturing both building and environmental influences. The method comprehensively quantified, compiled, summarized, and compared the architectural and environmental colors, offering practical universality. The findings aided Macao's color planning and urban renewal, presenting a novel urban color study approach. Gezawa et al. introduced a fused feature network that handled the shape classification and segmentation tasks by a dual-branch approach and feature learning. A feature encoding network was devised for network simplification by integrating two distinct building blocks with interposed batch normalization and rectified linear unit layers. It accelerated learning, mitigating gradient vanishing due to the limited number of layers for propagation. The framework also introduced a grid feature extraction module using convolution blocks and max-pooling to hierarchically represent input grid features. The max-pooling reduced the overfitting risk by gradually diminishing spatial dimensions, network parameters, and processing load. The grid size limitations were handled by locally sampling a constant point number from each grid region via a basic K-nearest neighbor by enhancing approximation functions for detailed feature characterization. It has shown superior performance with state-of-the-art techniques.

In another study, Ming et al. introduced deep CNN using CT scans for the diagnosis of severe pneumonia with pulmonary infection. An EC-U-net model has been employed on 120 patients to find accuracy in comparison to the traditional CNN. The learning rate of the model has decreased in over 40 training cycles by yielding results nearer to mask images. The given EC-U-net has outperformed the CNN with a higher Dice coefficient and lower loss. The method has increased diagnostic accuracy by reducing false rates and improving the recognition of infection-related features in CT scans by showing potential for clinical applications. Zhang et al. discussed a neural learning approach for the prediction of the best grasp configuration for each detected object from the image. A 3D-plane-based approach was used to filter the cluttered background and then the objects and grasp candidates by two separate branches were detected by an additional alignment module. A series of experiments are conducted on two public datasets to evaluate the performance of the proposed model in predicting reasonable grasp configurations “from a cluttered scene.” A deep learning-based method was proposed by Liu et al. to classify the data, screen out double-peak data, and realize the segmentation of the integral regions through the given U-Net segmentation model. The presented classification model exhibited an accuracy of 99.59%, while the segmentation model achieved an intersection over a union value of 0.9680 by using the combined loss function.

## 3. Conclusion

This editorial presented 10 research articles focused on the applications of Computational Intelligence for Signal and Image Processing. The aim was to gather related articles in the Signal and Image Processing industry, such as education, healthcare, and security. The findings presented in this Research Topic showcased more active development and research within the field of Computational Intelligence methods in the times ahead. To facilitate this progression, future approaches might encompass harnessing Computational Intelligence techniques to improve prediction precision and enhance the reliability of prediction models.

## Author contributions

DK: Data curation, Formal analysis, Investigation, Methodology, Project administration, Resources, Validation, Visualization, Writing—original draft, Writing—review and editing. BD: Conceptualization, Supervision, Visualization, Writing—review and editing.
